# Supplementary material for: MetaQTL: a package of new computational methods for the meta-analysis of QTL mapping experiments
Source: BMC Bioinformatics. 2007 Feb 8;8:49. doi: 10.1186/1471-2105-8-49 (PMC1808479; doi:10.1186/1471-2105-8-49)
Supplement: Additional File 7 — MetaQTL Package : jar file and tutorial. This Zip archive contains both the MetaQTL JAR file and the files of the tutorial. [file 1471-2105-8-49-S7.zip › org.inra.metaqtl/doc/index-files/index-4.html]

D-Index


|  |  |  |
| --- | --- | --- |
| **Overview** \| Package \| Class \| Use \| **Tree** \| **Deprecated** \| **Index** \| **Help** | |  |
| **PREV LETTER**   **NEXT LETTER** | **FRAMES**    **NO FRAMES**     **All Classes** |  |


A B C D E F G H I K L M N O P Q R S T U V W X Y Z 

---


## **D**

**d** - Variable in class org.metaqtl.MetaChrom: The absolute positions of markers in Morgan. **D** - Variable in class org.metaqtl.util.OffspringManager.HaldaneWaddingtonProportion: **D\_coeff** - Static variable in class org.metaqtl.util.OffspringManager.HaldaneWaddingtonProportion: **dataLayers** - Variable in class org.metaqtl.graph.MetaGraph: **dC** - Variable in class org.metaqtl.util.OffspringManager.HaldaneWaddingtonProportion: **dD** - Variable in class org.metaqtl.util.OffspringManager.HaldaneWaddingtonProportion: **dE** - Variable in class org.metaqtl.util.OffspringManager.HaldaneWaddingtonProportion: **DEFAULT\_HEIGHT** - Static variable in class org.metaqtl.graph.MetaGraphPar: The default height. **DEFAULT\_WIDTH** - Static variable in class org.metaqtl.graph.MetaGraphPar: The default width. **DEFINITION** - Static variable in interface org.thalia.bio.IOntologyTermProperties: The definition property of a term in a ontology **dF** - Variable in class org.metaqtl.util.OffspringManager.HaldaneWaddingtonProportion: **dfridr(NumericalUtilities.Function, double, double)** - Static method in class org.metaqtl.numrec.NumericalUtilities: Returns the derivative of a function func at a point x by Ridders' method of polynomial extrapolation. **dG** - Variable in class org.metaqtl.util.OffspringManager.HaldaneWaddingtonProportion: **DH** - Static variable in interface org.thalia.bio.IBioCrossTypes: **disableLogger()** - Method in class org.metaqtl.algo.MetaAlgorithm: **dist** - Variable in class org.metaqtl.TreeNode: The distance between the node and its parent. **distance(double, int, int)** - Static method in class org.metaqtl.util.MappingFunction: Returns the distance from the recombination rate. **distanceUnitCM** - Static variable in interface org.thalia.bio.entity.IGeneticMapProperties: **distanceUnitKey** - Static variable in interface org.thalia.bio.entity.IGeneticMapProperties: **dm** - Variable in class org.metaqtl.EMResult: The matrix of convergence rate for the EM-Algorithm (just for means). **DMAX(double, double)** - Static method in class org.metaqtl.numrec.NumericalUtilities: Returns the max between `a` and `b` **DO\_SEM** - Static variable in class org.metaqtl.algo.EMAlgorithm: If true the best starting point is used to do Supplemental EM (SEM) in order to compute the DM Matrix.. **doEM(double[], double[], int, EMResult)** - Static method in class org.metaqtl.algo.EMAlgorithm: Apply the EM-Algorithm on the given data set where `x` is an array of observed value and `sd` an array of same size than `x` which stores the standard deviations of the observed values. **DOF** - Static variable in interface org.metaqtl.IBioLGroupProperties: The key for the number of degrees of freedom. **dof** - Variable in class org.metaqtl.MetaChrom: The number of degrees of freedom. **dof** - Variable in class org.metaqtl.MultiFitResult: The number of degrees of freedom. **dominance** - Variable in class org.metaqtl.Qtl.QTLStats: The dominance effect of the QTL. **doProjection(ChromCluster[])** - Method in class org.metaqtl.algo.QtlProjAlgorithm: **doQtlClustering(MetaQtlData)** - Method in class org.metaqtl.algo.QtlClustAlgorithm: For a given data set `data` performs the clustering on each trait group and returns the results as an array of `MetaQtlResult` objects. **doTraitGroupClustering()** - Method in class org.metaqtl.MetaQtlData: According to the values of the QTLTrait.group attribute of the trait attribute of the qtls this methos clusterize the qtls in trait groups. **doTraitOntologyClustering(IBioOntology)** - Method in class org.metaqtl.MetaQtlData: According to the given trait ontology this methods clusterize the QTL in group in such a way that each group represents a trait term in the ontology. **draw(Graphics2D)** - Method in class org.metaqtl.graph.ChromLayer: **draw(Graphics2D)** - Method in class org.metaqtl.graph.ChromShape: **draw(Graphics2D)** - Method in class org.metaqtl.graph.GraphShape: Draws the shape. **draw(Graphics2D)** - Method in class org.metaqtl.graph.Layer: Draws the layer on the given `graph` context. **draw(Graphics2D, double, double)** - Method in class org.metaqtl.graph.MetaGraph: **draw(Graphics2D)** - Method in class org.metaqtl.graph.QtlLayer: **draw(Graphics2D)** - Method in class org.metaqtl.graph.QtlPartitionLayer: **draw(Graphics2D)** - Method in class org.metaqtl.graph.QtlShape: **draw(Graphics2D)** - Method in class org.metaqtl.graph.QtlTreeLayer: **DSWAP(double[], int, int)** - Static method in class org.metaqtl.numrec.NumericalUtilities: Swaps elements i and j in a. **DUBIOUS\_ORDER** - Static variable in interface org.metaqtl.IBioLocusProperties: The key for the marker dubious status. **DubiousLocusFactory** - Class in org.metaqtl.factory: A factory to read a list of dubious markers from a file. **DubiousLocusFactory()** - Constructor for class org.metaqtl.factory.DubiousLocusFactory

---


|  |  |  |
| --- | --- | --- |
| **Overview** \| Package \| Class \| Use \| **Tree** \| **Deprecated** \| **Index** \| **Help** | |  |
| **PREV LETTER**   **NEXT LETTER** | **FRAMES**    **NO FRAMES**     **All Classes** |  |


A B C D E F G H I K L M N O P Q R S T U V W X Y Z 

---
